# Lack of Autophagy Induction by Lithium Decreases Neuroprotective Effects in the Striatum of Aged Rats

**DOI:** 10.3390/pharmaceutics13020135

**Published:** 2021-01-21

**Authors:** Angelica Jardim Costa, Adolfo Garcia Erustes, Rita Sinigaglia, Carlos Eduardo Neves Girardi, Gustavo José da Silva Pereira, Rodrigo Portes Ureshino, Soraya Soubhi Smaili

**Affiliations:** 1 Department of Pharmacology, Universidade Federal de São Paulo, São Paulo-SP 04044-020, Brazil; angelicajardimcosta@gmail.com (A.J.C.); adolfo.erustes@gmail.com (A.G.E.); gustavo.pereira@unifesp.br (G.J.d.S.P.); ssmaili@unifesp.br (S.S.S.); 2 Electron Microscopy Centre, Universidade Federal de São Paulo, São Paulo-SP 04023-060, Brazil; rita.sinigaglia@unifesp.br; 3 Department of Psychobiology, Universidade Federal de São Paulo, São Paulo-SP 04023-062, Brazil; carlosnevesgirardi@gmail.com; 4 Departament of Biological Sciences, Universidade Federal de São Paulo, Diadema-SP 09913-030, Brazil; 5 Laboratory of Molecular and Translational Endocrinology, Escola Paulista de Medicina, Universidade Federal de São Paulo, São Paulo-SP 04039-032, Brazil

**Keywords:** aging, autophagy, lithium, striatum, mitochondria, lysosome

## Abstract

The pharmacological modulation of autophagy is considered a promising neuroprotective strategy. While it has been postulated that lithium regulates this cellular process, the age-related effects have not been fully elucidated. Here, we evaluated lithium-mediated neuroprotective effects in young and aged striatum. After determining the optimal experimental conditions for inducing autophagy in loco with lithium carbonate (Li_2_CO_3_), we measured cell viability, reactive oxygen species (ROS) generation and oxygen consumption with rat brain striatal slices from young and aged animals. In the young striatum, Li_2_CO_3_ increased tissue viability and decreased ROS generation. These positive effects were accompanied by enhanced levels of LC3-II, LAMP 1, Ambra 1 and Beclin-1 expression. In the aged striatum, Li_2_CO_3_ reduced the autophagic flux and increased the basal oxygen consumption rate. Ultrastructural changes in the striatum of aged rats that consumed Li_2_CO_3_ for 30 days included electrondense mitochondria with disarranged cristae and reduced normal mitochondria and lysosomes area. Our data show that the striatum from younger animals benefits from lithium-mediated neuroprotection, while the striatum of older rats does not. These findings should be considered when developing neuroprotective strategies involving the induction of autophagy in aging.

## 1. Introduction

There is a progressive loss of cellular homeostasis during aging, accompanied by a reduction in the progenitor cell reserve. These results were seen in many senescent cells that present organellar defects, ultimately leading to a gradual decline in function during aging [1,2]. The cells of the central nervous system (CNS), including the striatal neurons, are especially vulnerable to cell death during aging. Previous studies have associated this enhanced vulnerability to elevated reactive oxygen species (ROS) levels, reduced mitochondrial membrane potential (Ψm), increased pro-apoptotic Bax and/or reduced anti-apoptotic Bcl-2 protein expression [3,4].

Additionally, one of the most critical regulators of cell homeostasis is autophagy, which plays a vital role in longevity in several experimental models [5,6,7,8]. Autophagy is a highly regulated catabolic process that generally participates in cellular viability mechanisms under stress conditions, such as nutrient deprivation, hypoxia, endoplasmic reticulum stress, DNA damage, among others [9,10]. This process contributes to the renewal of cellular components related to dysfunctional organelles, macromolecules, long-lived and misfolded proteins, toxins and parasites [11,12]. The macroautophagy herein referred to as autophagy involves the formation of autophagosomes with a double-membrane structure, which fuses with lysosomes to form the autophagolysosomes where the cargo is degraded and recycled [11,13,14].

While numerous studies have associated age-related cellular homeostasis dysregulation with autophagy defects, it remains unclear how this catabolic process is modulated throughout life. It has been postulated that the accumulation of undigested material in the lysosomes (lipofuscin) and secondary lysosomes (e.g., autolysosomes) during aging could interfere with autophagosome fusion and cargo degradation, eventually leading to cell death [15] and favoring the pathogenesis of neurodegenerative processes [16,17]. In this context, the pharmacological modulation of autophagy represents a potential therapy for age-related diseases. Indeed, rapamycin [18], clonidine [19] and trehalose [20] have been shown to provide autophagy-mediated neuroprotection. Furthermore, Harrison et al. (2009) demonstrated that rapamycin increases the life expectancy of male mice by 9% and females by 14% [21].

Over the last twenty years, a growing amount of evidence shows that lithium could be used to treat neurodegenerative disorders [22,23,24,25,26]. For example, Sarkar et al. demonstrated that lithium induces autophagy, independently of mTOR, by inhibiting inositol monophosphatase (IMPase), resulting in the clearance of huntingtin and α-synuclein in non-neuronal and neural precursor cells [27]. On the other hand, studies have reported that lithium is a direct [28,29] and indirect [30,31] negative regulator of GSK3-β, contributing to autophagy inhibition by activating mTOR [32]; thus, an imbalance of these pathways can affect autophagy regulation. Since few reports have explored the modulation of autophagy in the aging striatum [33,34,35], the present study sought to evaluate the effects of lithium treatment on this brain structure in young and aged animals, which is particularly relevant to neurodegeneration.

## 2. Materials and Methods

### 2.1. Animals

The experiments were performed with two groups of Wistar 2BAW rats, young (4–6 months-old) and aged (24–26 months-old). The sample size (*n* = 2–4) was determined based on the reproducibility of the results and considering the challenges of keeping aged animals for the proposed work. It is important to point out that during the process of aging, 2/3 of the animals died and only the animals that reached the age of 24–26 months were considered. All the samples and biologic materials available were studied, where a maximum number of images and paramethers were acquired and measured. Thus, this provided reproducible and unique results in terms of aging studies. Throughout the experimental period, the animals were maintained in the Institute of Pharmacology and Molecular Biology animal facility at the Federal University of São Paulo, under controlled light (12 h light–dark cycle) and temperature (23 ± 2 °C) conditions to assure the quality control and the accuracy of the experiments. The rats consumed standard rat chow and water ad libitum. All the procedures performed in this study were approved by and are in accordance with the ethical standards of the Institutional Ethics Committee (CEUA/UNIFESP 1907220814).

### 2.2. Acute and in Loco Treatments in Striatal Slices

#### 2.2.1. Brain Slices Preparation and Lithium Treatments

Tissue collection involved euthanizing the animals by decapitation, gently opening the skulls, and quickly removing the whole brain. Each brain was then gently sliced with a vibratome. The subsequent coronal sections containing the frontal cortex, corpus callosum and striatum triad of approximately 400 µm were immersed in an artificial cerebral–spinal fluid (aCSF) solution supplemented with sucrose (2 mM KCl, 1 mM CaCl_2_, 26 mM NaHCO_3_, 1.25 mM NaH_2_PO_4_, 1 MgCl_2_, 2 mM MgSO_4_, 10 mM glucose, sucrose 248 mM, pH 7.4). Then the slices were incubated in maintenance aCSF solution (2 mM KCl, 2 mM CaCl_2_, 26 mM NaHCO_3_, 1.25 mM NaH_2_PO_4_, 2 mM MgSO_4_, 10 mM glucose, 124 mM NaCl, pH 7.4) [4]. The solution was constantly aerated with a perfusion pump and maintained at room temperature (20–25 °C). The slices were kept under these conditions for at least 45 min for tissue stabilization and then treated with lithium carbonate (Li_2_CO_3_, at concentrations of 0.1, 1 or 10 mM) in the presence or absence of ammonium chloride (NH_4_Cl). In all experiments the untreated striatal slices of either young (4–6 months-old) or aged (24–26 months-old) animals were used as controls.

#### 2.2.2. Western Blotting Analysis

Coronal sections of the striatum were dissected and homogenized in radioimmunoprecipitation assay (RIPA) buffer (150 mM NaCl, 1% NP-40, 0.5% sodium deoxycholate, 0.1% SDS, 50 mM Tris-HCl, 2 mM MgCl_2_) supplemented with protease inhibitors (Phenylmethylsulfonyl fluoride—PMSF and Protease Inhibitors Cocktail). The homogenate was sonicated and centrifuged at 5000 rpm for 30 min at 4 °C to remove the cellular debris, and the supernatant fraction was collected. An aliquot of the supernatant corresponding to 50 µg of total protein was subjected to thermal denaturation (95 °C for 10 min) in sample buffer (12% SDS, 40% glycerol, 120 mM ethylenediaminetetraacetic acid (EDTA), 1 mg/mL bromophenol blue, 375 mM Tris/HCl, pH 6.8). The protein samples were then separated by SDS-PAGE and subsequently transferred to a polyvinylidene difluoride (PVDF) membrane (Merck Millipore, Burlington, MA, USA). The membranes were probed with the following primary antibodies: rabbit polyclonal anti-Ambra 1 (1:200, Merck Millipore, Burlington, MA, USA, Cat# ABC131), rabbit monoclonal anti-beclin-1 (1:1000, Cell Signaling Technology, Danvers, MA, USA, Cat# 3738), rabbit monoclonal anti-LC3B (1:1000, Cell Signaling Technology, Cat# 2775), rabbit monoclonal anti-p-GSK-3β (1:500, Cell Signaling Technology, Cat# 9336), rabbit monoclonal anti-GSK-3β (1:1000, Cell Signaling Technology, Cat# 9581), rabbit monoclonal anti-LAMP1 (1:500; Santa Cruz Biotechnology Inc, Dallas, TX, USA, Cat# sc-20011) and, as an internal control, rabbit polyclonal anti-β-actin (1:1000; Sigma-Aldrich, St. Louis, MO, USA, Cat# A5060). After overnight incubation, the membranes were washed and then incubated with the appropriate Horseradish Peroxidase-conjugated secondary antibodies, goat polyclonal anti-rabbit (1:5000; Thermo Fisher Scientific, Waltham, MA, USA, Cat# 31460) and goat polyclonal anti-mouse (1:5000; Thermo Fisher Scientific, Cat# G-21040). The Western blots were developed with enhanced chemiluminescent reagent (PerkinElmer, Waltham, MA, USA) and the immunoreactive bands were visualized using a high-resolution photo documentation system (UVITEC Alliance 4.7, Cambridge, UK). The semiquantitative analysis was performed by densitometry evaluating optical intensity of the immunoreactive protein bands using the Alliance software (UVITEC, Cambridge, UK). The intensity of the bands was normalized in relation to the β-actin band. All data were expressed as a percentage of the young control group (100%).

#### 2.2.3. Cell Viability and ROS Measurements

The cell viability and ROS generation assays were performed in a multi-mode microplate reader (FlexStation 3, Molecular Devices, San Jose, CA, USA) operating in the fluorescence mode. The fluorescence data were extracted using the SoftMax Pro 7 software (Molecular Devices).

For the cell viability assay, the lithium-treated and untreated brain slices of young and aged rats were loaded with 10 µM Calcein-AM (excitation/emission: 488/525 nm) plus 0.01% Pluronic F-127 for 30 min at 37 °C. Calcein-AM, when crossing the cell membrane, is cleaved by esterases of viable cells producing a fluorescent compound. The fluorescence intensity was recorded, and the maximum fluorescence was used for the analyses.

For the measurements of ROS generation, the lithium-treated and untreated brain slices of young and aged rats were incubated with 5 µM CM-H_2_DFCDA (“5-(and-6-)-chloromethyl-2′,7′-dichlorodihydro fluorescein diacetate, acetyl ester”) indicator (ex./em. 488/525 nm) for 30 min at 37 °C. CM-H_2_DFCDA in the presence of reactive species is oxidized to 2′,7′-dichlorofluorescein (DCF), a highly fluorescent product. Initially, a baseline was recorded for 2 min, and then the slices were stimulated with 1 mM glutamate. Metabotropic and ionotropic receptor stimulation by glutamate leads to an increase in cytoplasmic Ca^2+^ level, which can activate the electron transport chain and increase ΔΨm and ROS generation [36]. The tissues were then treated with 60 mM tert-butyl hydroperoxide (TBHP), which produces the maximum fluorescence (Fmax) by promoting an increase in ROS production that oxidizes all of the intracellular DCF. The values were normalized to the baseline fluorescence and expressed as percentages of the maximum fluorescence (Fmax = 100%).

#### 2.2.4. Oxygen Consumption Rate Measurement

The coronal brain slices were treated with lithium as described above, and then oxygen consumption was measured. Striatal slices were isolated to assess the mitochondrial respiration rate using an oxygraph system (OXYG2, Hansatech Instruments, Leicester, UK). Each striatal slice was immersed in the oxygraph chamber containing aCSF and maintained at 37 °C. The baseline oxygen consumption values were recorded for 2.5 min, followed by the addition of adenosine diphosphate (ADP, 5 µM) and inorganic phosphate (Na_2_HPO_4_, 5 µM). These substrates were used to stimulate mitochondrial respiration state 3, with a subsequent increase in oxygen, which should return to the basal state, state 4, after approximately 5 min. The oxygen consumption was expressed as a linear regression function of its concentration over time. The rate of state 3/4 was considered as the ADP:O ratio (O_2_ consumption rate per ADP molecule consumed). Basal oxygen consumption was established by state 4 measures and data expressed as a percentage of the young control samples (100%).

### 2.3. Chronic and In Vivo Treatment and Ultrastructural Analysis

For the ultrastructural analysis of the striatum, two animals from each group were treated with Li_2_CO_3_ in the drinking water (4.7 mM) given ad libitum for 30 days. After treatment, all animals were anesthetized with halothane, intubated and connected to a mechanical respirator. The animals were then transcardially perfused with phosphate-buffered saline (PBS) solution containing 2.5% glutaraldehyde and 2.0% formaldehyde at a continuous flow of 15 mL/min using a perfusion pump (Easy-load model 7518-60, Masterflex pump controller, Cole Parmer, Vernon Hills, IL, USA). After euthanizing, the brains were gently removed and left overnight in the same fixative. The brains were coronally cutted at 1 mm-thick. One section from the dorsolateral region of the striatum (1.8 mm from bregma, Paxinos) was trimmed for obtaining a 1-mm^3^ sample. The samples were washed in 0.1 M cacodylate buffer and post-fixed in 2% osmium tetroxide/3% potassium ferrocyanide (30 min), contrasted in uranyl acetate, dehydrated in alcohol, cleared in propylene oxide and embedded in Epon resin. Semi-thin sections (500 nm thick) were obtained using an ultramicrotome (Ultracut R, Leica, Wetzlar, Germany) and stained with toluidine blue alcoholic solution. These semi-thin sections were examined under a light microscope (Olympus BX41) to select the optimal area to be observed in a transmission electron microscope. We then obtained ultrathin sections (70 nm thick) using an ultramicrotome (Ultracut R, Leica). These sections were collected in copper mesh, contrasted with uranyl acetate and lead citrate, and subsequently examinated with a transmission electron microscope (1200 EXII, JEOL). Electron micrographs were obtained with a Multiscan 791 digital camera (GATAN, Pleasanton, CA, USA), and the obtained images were analyzed with the ImageJ software (1.46r, NIH, Bethesda, MD, USA).

### 2.4. Statistical Analyses

All values are represented as the mean ± standard error of the mean (SEM). Comparisons between two means were determined using the unpaired Student’s *t* test. Between group comparisons (three or more means) were performed using one-way ANOVA followed by Dunnett’s post-hoc test and two-way ANOVA followed by Bonferroni’s post-hoc test using the GraphPad Prism 5 software. Differences were considered statically significant when *p* < 0.05.

## 3. Results

### 3.1. Acute Lithium Treatment Promotes Opposite Effects on Autophagy in Young and Aged Rat Striatum

**Figure 1:**
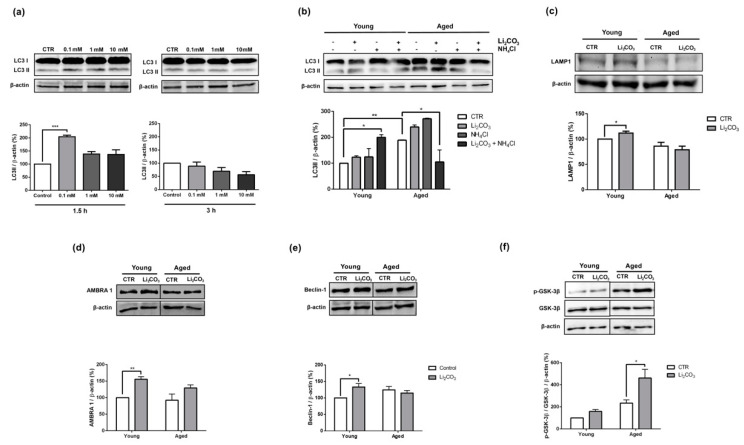
Lithium blocked autophagy in striatal slices of aged rats, possibly by GSK-3β inhibition. (**a**) Young animals striatal slices were treated with several concentrations (0.1, 1 and 10 mM) of Li_2_CO_3_ for 1.5 h and 3 h, and the LC3-II expression was detected by Western blotting. (**b**) LC3-II expression levels were detected by Western blotting after 1.5 h of 0.1 mM Li_2_CO_3_ treatment in the presence or absence of NH_4_Cl (4 mM) in young and aged animals. (**c**) LAMP 1, (**d**) Ambra 1, (**e**) Beclin-1, (**f**) p-GSK-3β (Ser 9) and GSK-3β expressions levels were detected by Western blotting after 1.5 h of 0.1 mM Li_2_CO_3_ treatment in striatal slices of young and aged animals. Representative Western blot autoradiographies are shown on the top panels and the histograms showing mean ± standard error of the mean (SEM) of bands’ relative density are shown on the bottom panels. All values were normalized to β-actin and expressed as percentages of the control young animals (100%) (*n* = 2–4). (**a**) * *p* < 0.05 compared to control (one-way ANOVA, Dunnet post-hoc); (**b**,**d**–**f**) * *p* < 0.05 or ** *p* < 0.01 compared to control (one-way ANOVA, Bonferroni post-hoc); (**c**) * *p* < 0.05 compared to control (Student’s *t*-test).

Before comparing the effects of lithium on young and aged striatal slices, we first determined the concentration and exposure time required for inducing autophagy in the young samples, as evidenced by an increase in LC3-II expression. As shown in Figure 1a, striatal slices from young rats were treated in loco with three different concentrations of Li_2_CO_3_ (0.1 mM, 1 mM and 10 mM) for 1.5 h or 3 h. We found that 0.1 mM of Li_2_CO_3_ for 1.5 h induced an increase in LC3-II levels compared to the untreated control slices. Notably, this effect was no longer evident after 3 h. Since lithium appeared to be a potential autophagic inducer, we then evaluated the effect of treating striatal slices from aged rats with 0.1 mM of Li_2_CO_3_ for 1.5 h.

Then, we evaluated LC3-II levels in striatal slices from young and aged rats treated in loco with 0.1 mM of Li_2_CO_3_ for 1.5 h in the presence (added in the last hour of the Li_2_CO_3_ treatment) or absence of 4 mM ammonium chloride (NH_4_Cl). This weak base is protonated in acidic organelles and inhibits the terminal stages of autophagy. This methodology provides invaluable information about the kinetics of the system and cargo clearance, assessing LC3 II turnover [37]. Figure 1b shows that the striatal slices from the aged rats presented significantly higher basal levels LC3-II than the young control slices. In the striatal slices from young rats, Li_2_CO_3_ induced the complete autophagic flux, and Li_2_CO_3_ + NH_4_Cl resulted in a nearly two-fold increase in LC3-II levels compared to Li_2_CO_3_ alone. On the other hand, striatal slices from aged rats treated with Li_2_CO_3_ + NH_4_Cl exhibited a significant decrease in the levels of LC3-II, a result that suggests that autophagy might be impaired in the aged animals (Figure 1b).

LAMP1 expression was also assessed in striatal slices of young and aged animals treated with Li_2_CO_3_ for 1.5 h. LAMP 1 is a structural protein of the lysosome/late endosome that can be used as another indicator for autophagic flux modulation [37]. The results showed that treatment with Li_2_CO_3_ increased the expression of LAMP 1 in young animals. Furthermore, it is possible to observe a reduction in the LAMP 1 expression of aged animals compared to the young control group, although no statistical significance was detected (Young CTR vs Aged CTR *p* value = 0.1394) (Figure 1c).

Since the induction of autophagy is mainly regulated by the Beclin 1 complex, which interacts with several cofactors such as Ambra 1 (activating molecule in Beclin-1-regulated autophagy) [38], we investigated the effects of Li_2_CO_3_ treatment on Ambra 1 and Beclin-1 expression levels (Figure 1d,e) in the striatum of young and aged rats. In the young animal slices treated with Li_2_CO_3_, the levels of both Ambra 1 and Beclin-1 were significantly increased when compared to the young control group. While a similar trend was also observed in the striatal slices of aged rats treated with Li_2_CO_3_, these values failed to reach a level of significance and thus indicate no modulation in the levels of these autophagy-inducer phase proteins (Figure 1d,e). Next, we evaluated the effect of Li_2_CO_3_ treatment on glycogen synthase kinase 3β phosphorylation. For this, we utilized an antibody that specifically reacts with the inactive form of GSK-3β phosphorylated at serine 9 (p-GSK-3β, a posttranslational modification related to the mTORC1 inactivation and autophagy induction). Here we observed that the expression levels of p-GSK-3β were significantly increased in brain slices of aged rat treated with Li_2_CO_3_ (Figure 1f). This result suggests that inhibition of the GSK-3β pathway may be involved in autophagy blockage in older animals.

### 3.2. Acute Lithium Treatment Exerts an Age-Dependent Effect on Cell Viability and ROS Generation

**Figure 2:**
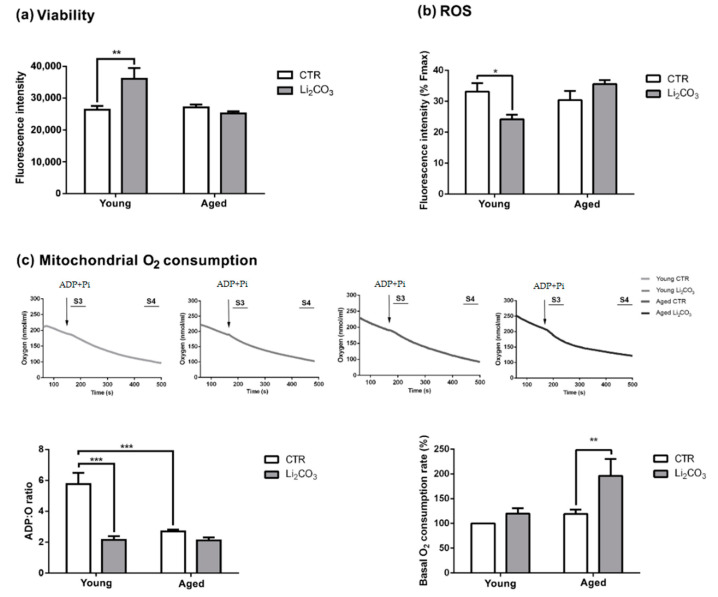
Cytoprotective effect of lithium in young but not aged striatal slices. Histograms of the mean maximum fluorescence of (**a**) calcein in the striatum of young and aged rats, (**b**) 5-(and-6-)-chloromethyl-2′,7′-dichlorodihydrofluorescein diacetate, acetyl ester (CM-H_2_DFCDA) after stimulus with glutamate (1mM) and normalized by tert-butyl hydroperoxide (TBHP) (30 mM); (**c**) Histogram of the adenosine diphosphate (ADP):O ratio (state 3/4) after the addition of adenosine diphosphate (ADP, 5 µM) and inorganic phosphate (Na_2_HPO_4_, 5 µM) and basal oxygen consumption rate analyzed by state 4 measures. The values are presented as a ratio or percentage of consumption oxygen rate (**b**,**c**). All histograms are a mean ± SEM (*n* = 4). * *p* < 0.05, ** *p* < 0.01 and *** *p* < 0.001 compared to control (two-way ANOVA, Bonferroni post-hoc).

After assessing the effect of Li_2_CO_3_ on autophagy induction, we then decided to focus our attention on the possible role this ion has in cytoprotection. Towards this goal, we evaluated the effects of Li_2_CO_3_ on cell viability (Figure 2a) and ROS generation (Figure 2b) in the striatum from young and aged rats by measuring the maximal fluorescence emitted from Calcein-AM and CM-H_2_DFCDA, respectively. As shown in Figure 2a, the Li_2_CO_3_-treated striatal slices from the young rats exhibited significantly higher fluorescence intensities, indicating enhanced cell viability compared to untreated control slices. In contrast, Li_2_CO_3_ did not affect the cell viability of the striatal slices from aged animals (Figure 2a).

The quantification of the CM-H_2_DFCDA fluorescence, following glutamate stimulation, demonstrated that Li_2_CO_3_ attenuates ROS production in the striatal slices of young rats when compared to untreated control slices. On the other hand, Li_2_CO_3_ afforded no protection against the glutamate-induced ROS generation in the brain slices from aged animals (Figure 2b).

### 3.3. Acute Lithium Treatment Increases O_2_ Consumption

The mitochondrial respiratory function was assessed by measuring the ADP:O ratios and basal O_2_ consumption rates in the striatum slices of young and aged animals treated with 0.1 mM Li_2_CO_3_ for 1.5 h. We observed that the untreated control brain slices from the young rats presented a significantly higher ADP:O ratio than aged control slices. Interestingly, young striatal slices treated with Li_2_CO_3_ displayed a significantly reduced ADP:O ratio comparable to the ratios observed in the untreated and Li_2_CO_3_-treated slices from aged animals (Figure 2c, bar graph, lower left). Concerning the basal rate of O_2_ consumption, a significantly higher value was observed in the aged striatal slices treated with Li_2_CO_3_, suggesting that mitochondrial respiration is modulated under these conditions (Figure 2c, bar graph, lower right).

### 3.4. Chronic Lithium Treatment Increases Organelle Damage in Aged Striatum

To determine if lithium induces ultrastructural changes in the striatum, we administered Li_2_CO_3_ to young and aged animals for 30 days. The consumption of lithium solution (4,7 mM Li_2_CO_3_, dissolved in drinking water—which corresponds to 0.35 mg/mL) was monitored during the 30 days of treatment to obtain an average dose administered per animal. The young animals consumed an average of 20 ± 3.7 mL/day of Li_2_CO_3_ solution, and considering the animal weight of approximately 208.8 ± 8.5 g, it corresponds to ≈34 mg/kg. Likewise, the aged animals consumed 24.8 ± 6.9 mL/day of Li_2_CO_3_ solution and presented an average weight of 222.6 ± 34.9 g, resulting in a calculated dose of ≈38 mg/kg. Therefore, an average of 4 mg/kg more was administered to the aged animals; however, considering that the weight and lithium consumption of aged animals are less homogenous than in the young, this difference does not represent a significant mean variation in lithium uptake between groups.

**Figure 3:**
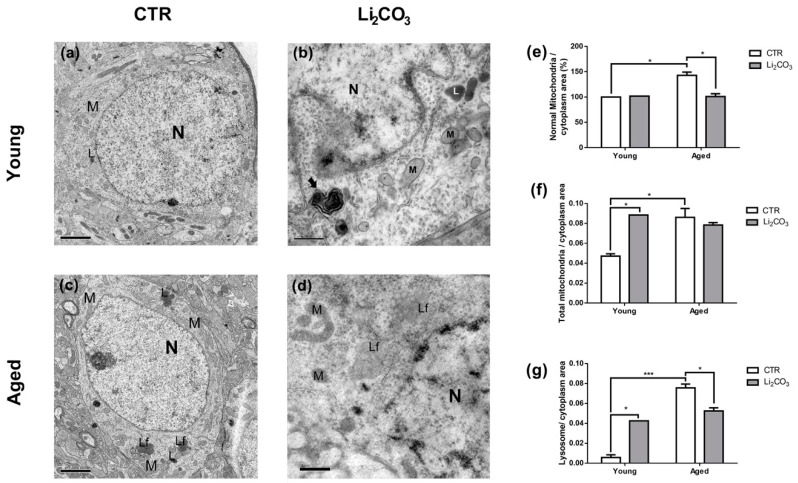
Lithium alters the morphology of organelles in the striatum of aged rats. Representative electron micrographs of dorsolateral striatal neurons from young and aged control animals (**a**) and (**c**), respectively) or Li_2_CO_3_ treated (4.7 mM, 30 days) young and aged animals (**b**) and (**d**), respectively). In (**a**,**c**), representative striatal neurons displaying diverse organelles with normal appearances, like mitochondria (M), lysosome (L), telolysosome (lipofuscin granule, Lf) and nucleus (N). In (**b**), a striatal neuron of a Li_2_CO_3_-treated young animal showing the normal ultrastructural appearance of all organelles like mitochondria (M), heterolysosome (L), and nucleus (N) and the presence of an autophagic vacuole (arrow). In (**d**), a striatal neuron of a Li_2_CO_3_-treated aged animal revealing some telolysosome (lipofuscin granule, Lf) and electrondense mitochondria with swollen cristae (M). Scale bars represent 2 µm in (**a**) and (**b**); 1 µm in (**c**) and (**d**). Micrographs digitally obtained (Orius GATAN, USA) through a transmission electron microscope at 80 kV (JEOL1200 EXII, Japan). Histograms showing normal (**e**) and total (**f**) mitochondria area, and lysosomes (**g**) in relation to the cytoplasm area. In (**e**) the values are presented as a percentage of the control. All histograms are a mean ± SEM (*n* = 2). * *p* < 0.05 and *** *p* < 0.001 (two-way ANOVA, Bonferroni post-hoc).

In Figure 3a, the striatum from young untreated control animals exhibits the typical ultrastructural features of medium spiny neurons [39], a typical area of mitochondria (Figure 3e) and a small number of lysosomes (Figure 3g). In contrast, there were significantly more normal and total mitochondria area per cytoplasm area (Figure 3e,f) and larger lysosomes, many filled with lipofuscin (telolysomes) in the untreated control striatum from aged rats (Figure 3c). The representative image of Li_2_CO_3_-treated striatum from a young rat in Figure 3b shows the normal ultrastructural appearance of all organelles and the presence of autophagic vacuoles (arrow) (Figure 3b). In contrast, total mitochondria and lysosomes area were increased (Figure 3f,g). Treating aged striatum with Li_2_CO_3_ caused alterations in mitochondria, including reduced normal mitochondria area (Figure 3e), swelling and disarranged cristae (Figure 3d). Furthermore, the number and size of the lysosomes were significantly reduced in Li_2_CO_3_-treated aged striatum (Figure 3d,g), which corroborates our previous findings with the autophagy markers.

## 4. Discussion

Several studies have demonstrated that lithium treatment reduces neurodegeneration parameters in experimental models [23,40,41,42,43,44,45,46,47,48]. However, most studies on the protective actions of lithium were performed with cell lines or young animals. In the present study, we sought to determine if lithium exerts age-dependent effects that could differentially modulate autophagy, a catabolic protective strategy against neurodegenerative diseases. Towards this goal, we evaluated mitochondrial alterations, including morphological changes, ROS generation and oxygen consumption in young and aged striatum treated with lithium.

It is well known that autophagic activity decreases with age, and it has been shown that impaired autophagy contributes to neurodegeneration in mice [17,49]. Here we demonstrated that lithium modulates autophagy in a brain structure which is a target to the neurodegenerative process in an age-dependent manner. For example, we showed that the levels of LC3-II are elevated in aged striatum. However, upon the treatment with a concentration of Li_2_CO_3_ that induces autophagy, the LC3-II levels drop sharply (Figure 1b). Additionally, LAMP 1 levels were reduced in untreated and treated aged striata (Figure 1c), suggesting that autophagy flux was blocked. This observation agrees with the results reported by Yamamoto et al. in the murine kidney, where they observed higher basal autophagic activity in aged samples. However, when the young and aged animals were submitted to starvation protocol, only the kidneys of the young animals exhibited GFP-LC3B positive puncta [50], providing evidences that autophagic flux was blocked in older animals. Moreover, Liu et al. (2019) detected higher Beclin-1 and LC3-II expression levels in the striatum of aged rats. They also found that regular aerobic exercise regulates the balance between cell death and autophagy [33].

Lithium is commonly used to treat psychiatric disorders, but the precise mechanism of action is a subject of intense debate in the literature. It has been reported that lithium can modulate autophagy, either in an mTOR dependent or independent pathways, by inhibiting IMPase. Moreover, it is well known that lithium inhibits GSK3-β, but the role this kinase plays in autophagy is controversial. For example, in the MCF-7 breast cancer cell line, inhibition of GSK-3β increases autophagy by negatively modulating mTORC1 [51]. On the other hand, in neurodegeneration, Sarkar et al., 2008 demonstrated that lithium increased autophagy by inhibiting IMPase, but decreased autophagy by inhibiting GSK-3β [32]. Here, in aged striatum treated with lithium, we observed higher p-GSK-3β phosphorylation levels at an inhibitory site, which resulted in autophagy blockage (Figure 1f).

Additionally, in contrast to the young striatum, the expression of autophagy inducer-phase proteins Ambra 1 and Beclin-1 were unaltered in the aged tissue, reinforcing the idea that autophagy is inhibited, despite the presence of an autophagy inducer. Interestingly, Castillo-Quan et al., 2016 demonstrated that lithium increased the life and health spans in a Drosophila melanogaster aging model. The authors proposed that these effects are probably due to GSK-3β pathway inhibition and do not involve autophagy [52].

Previous studies have also shown that the accumulation of insoluble particles known as lipofuscins can obstruct the autophagic system, leading to an autophagy deficiency in the aging CNS [15,53,54]. Indeed, in this work, we demonstrated that striatum from aged animals presented more lysosomes, with many filled with lipofuscin (Figure 3c,g). Surprisingly, chronic Li_2_CO_3_ treatment reduced the area of lysosomes in the age-matched group (Figure 3d,g). Therefore, since the autophagic flux is attenuated in the Li_2_CO_3_-treated aged striatum (Figure 1b), it is plausible that the lower area of lysosomes and LAMP 1 levels can contribute to dysregulation in autophagy. However, we can not exclude that a decreased number of lysosomes could be associated to a non-apoptotic cell death triggering [55].

On the other hand, lithium in therapeutic formulations is more related to neuronal survival in several animal models of neurodegeneration, including focal cerebral ischemia, 3-NP, quinolinic acid-induced Huntington’s disease-like pathogenesis and a Down’s syndrome model [23,40,41,42]. Fabrizi et al., 2014 demonstrated that lithium promoted cell proliferation and survival and protected PC12 cells (pheochromocytoma cell line) against trimethyltin -induced apoptosis [56]. In this sense, our results agree with these findings since Li_2_CO_3_ was able to increase tissue viability in the young striatum (Figure 2a) but not in aged animals. More evidence of lithium-mediated neuroprotection being age-dependent comes from a study using a transgenic Alzheimer’s disease model, in which the drug significantly improved neurogenesis and memory in two-month-old animals compared to six-month-old animals [57]. Autophagy inhibition may be involved with these effects in aged animals; however, it may not be the only factor involved since autophagy blockers did not increase cytotoxicity in vitro [55].

Kim et al., 2011 demonstrated the protective effects of lithium in neurons, after the induction of oxidative stress in models of Parkinson’s disease, both in vitro (dopaminergic neurons overexpressing the A53T mutant of α-synuclein) and in vivo (transgenic mice overexpressing the A53T mutant of α-synuclein) [45]. Our results showed that Li_2_CO_3_ reduced ROS generation in young striatum but failed to elicit this effect in the aged group (Figure 2b). This result suggests a positive correlation with the increased tissue viability observed in the Li_2_CO_3_-treated young tissues (Figure 2a). Indeed, previous studies have shown that therapeutic concentrations (0.5–1 mM) of lithium exert neuroprotective effects due to increased protection against oxidative stress [45,58,59]. The increase in the basal O_2_ consumption rate may lead to enhanced ROS production in the samples treated with lithium (Figure 2c).

Remarkable metabolic alterations that are often associated with mitochondrial dysfunction accompany aging [60,61]. Indeed, alterations in glycolytic intermediates and the tricarboxylic acid cycle, which indicate mitochondrial dysfunction, have been observed during aging and in neurodegenerative diseases, such as Alzheimer’s [60]. Chen et al. showed that lithium treatment inhibits GSK-3β, reducing the inactivation of pyruvate dehydrogenase E1 (PDH-E1) and consequently increasing basal and maximal mitochondrial respiration [62]. Here we show that aging promotes a reduction in mitochondrial respiration function, as shown by the ADP:O ratio (Figure 2c), which could account for the mitochondrial alterations detected by electron microscopy. In an animal model of Amyotrophic Lateral Sclerosis, Fornai et al., 2008 showed that lithium reduced mitochondrial swelling and increased the number of normal mitochondria [63]. Here we show that chronic Li_2_CO_3_ treatment increased the total mitochondria area in young animals, as well as reduced normal mitochondria area in the striatum of aged animals, suggesting that lithium induces different subcellular alterations during aging. These increases in normal and total mitochondria area observed in aged animals may be related to autophagy/mitophagy inhibition. Previous studies have shown that autophagic inhibitors such as Wortmannin, bafilomycin A_1_ and chloroquine increased the mitochondrial mass in glioma and hepatocellular carcinoma cells lines [64,65]. Additionally, a study using aged rabbits (2.5 years old) with heart failure demonstrated an accumulation of proteins from the autophagic signaling cascade (LC3 and p62) and specific mitophagy processes (Parkin) associated with impairment of the mitochondrial fusion–fission process. It thus could increase the mitochondrial mass [66].

Considering all of the results and discussion in the present study, we conclude that Li_2_CO_3_ treatment may benefit younger animals by attenuating ROS production, modulating autophagic activity and augmenting tissue viability. However, Li_2_CO_3_-treated aged animals did not present these cytoprotective effects, in addition to lithium-mediated autophagy inhibition. Thus, autophagy appears to be an essential factor for neuroprotection in the young striatum. Indeed, our data show that aged animals present autophagy deficiencies that could limit the putative Li_2_CO_3_-induced actions. We expect that future studies will identify new autophagy-inducing targets that provide neuroprotection in older animals.

## Data Availability

The data presented in this study are available on request from the corresponding author. The data are not publicly available due to privacy.
